# Home injury prevention attitude and performance: a community-based study in a WHO safe community

**Published:** 2019-08

**Authors:** Mohammad Saadati, Jafar Sadegh Tabrizi, Ramin Rezapour, Riaz Alaei Kalajahi

**Affiliations:** ^*a*^Road Traffic Injury Research Center, Tabriz University of Medical Sciences, Tabriz, Iran.; ^*b*^Tabriz Health Services Management Research Center, Health Management and Safety Promotion Research Institute, Tabriz University of Medical Sciences, Tabriz, Iran.; ^*c*^Iranian Center of Excellence in Health Management, School of Management and Medical Informatics, Tabriz University of Medical Sciences, Tabriz, Iran.; ^*d*^Department of Health Management and Economics, School of Public Health, Tehran University of Medical Sciences, Tehran, Iran.

## Abstract

**Background:**

Unintentional injuries in the home are one of the threats to childhood quality of life which is considered as a social determinant of health. Regarding mother s leading role in taking care of the children in Iranian families, the present study was conducted to investigate mothers home-injury prevention attitude and performance and its contributing factors in Sahand, Iran.

**Methods:**

This was a cross-sectional study conducted in 2017. Sampling was done using convenience sampling method among all mothers of children less than five years old who attended the health centers to receive child care services. A valid attitude questionnaire and safety performance checklist were used for data collection. Data were analyzed through SPSS-24 software using descriptive (Frequency, mean, etc.) and inferential statistics (chi-square, Kruskal-Wallis) method.

**Results:**

The average age of mothers was 30.58 (±5.01). About 65% of the mothers held high school diplomas or lower degrees. The mean score of mothers' attitude was calculated to be 72.12(±6.79). More than 58% of the mothers had an appropriate level of attitude. The mothers' injury prevention performance mean score was 66.59 (±12.85). Family socioeconomic status, Mother’s age, educational level, and job, father s job, age and gender of the child were the contributing factors (p<0.05).

**Conclusions:**

Most of the mothers (about 59%) had an appropriate level of home-injury prevention attitude. However, the mothers' injury prevention performance was not at an acceptable level. It is necessary to employ educational and cultural interventions in the community to lead Iranian mothers, especially mothers with sons, adopt a safe behavior.

**Keywords:**

Attitude, Performance, Home Injuries, Mothers

